# Body art among Saudi youth: a cross-sectional study of knowledge, practices, and health risks

**DOI:** 10.3389/fmed.2026.1773903

**Published:** 2026-06-04

**Authors:** Hend M. Al-Atif, Norah Saad Jadaan, Saifaleslam A. Mahmoud, Faisal S. Alyahya, Almaha H. Alshathri, Anwar Mustafa Alsaeed, Aljohara H. Alshathri, Ruba Al Murayyi, Shadan Ali Al.Atif, Ravi Shankar Reddy

**Affiliations:** 1 Department of Internal Medicine, College of Medicine, King Khalid University, Abha, Saudi Arabia; 2 General Medicine Practice Program, Batterjee Medical College, Aseer, Saudi Arabia; 3 College of Medicine, King Abdulaziz University, Jeddah, Saudi Arabia; 4 Department of Family Medicine, Riyadh Second Health Cluster, Riyadh, Saudi Arabia; 5 Dermatology program, Almoosa Specialist Hospital, Ahsa, Saudi Arabia; 6 Department of Medical Rehabilitation Sciences, College of Applied Medical Sciences, King Khalid University, Abha, Saudi Arabia

**Keywords:** body piercing, health risks, knowledge, practices, Saudi Arabia, survey, tattoos, young adults

## Abstract

**Background:**

Body art, including body piercing and tattooing, has evolved from cultural and religious practices to mainstream forms of self-expression. This trend is evident globally and particularly among young adults in Saudi Arabia, where traditional values are being influenced by globalization and digital media. This study aims to assess awareness, attitudes, and practices regarding body piercing and tattooing among young adults in Saudi Arabia.

**Methods:**

A cross-sectional study was conducted using an online self-administered questionnaire distributed to young adults who have undergone body piercing and/or tattooing across all regions of Saudi Arabia. The study employed a final sample of 1,472 participants, calculated to account for a 20% non-response rate. Findings may not generalize to rural populations due to the online sampling method. Descriptive and inferential statistical analyses were performed using SPSS version 28.0.

**Results:**

The majority of participants (77.31%) were aware of health risks related to body art, though only 37.37% recognized non-infectious complications. Safety concerns were prevalent, with 77.45% doubting the hygiene standards of body art procedures. Ear piercings were the most common (83.69%), while tattoos and other piercings were less frequent. Interest in future body art was low among those without prior experience. Significant associations were found between age, gender, and perceptions of body art risks and practices.

**Conclusion:**

The study highlights a significant awareness gap regarding non-infectious risks associated with body art among young adults in Saudi Arabia. There is an urgent need for targeted public education and stricter regulation to ensure safe practices. The findings suggest that while awareness of risks exists, further education and improved safety standards are crucial to addressing the evolving trends in body art practices.

## 1 Introduction

Body art, including body piercing and tattooing, has evolved from a niche cultural practice to a widespread trend, particularly among young adults worldwide. Body modifications, historically rooted in cultural, religious, and tribal traditions, have served as symbols of social status, identity, and rites of passage. In recent years, these practices have transcended cultural boundaries and become mainstream forms of self-expression, particularly among younger demographics (1–3). The rise of social media and the influence of global fashion trends have further accelerated the acceptance and popularity of body art, embedding it in youth culture worldwide.

In Saudi Arabia, where cultural and religious values traditionally guide social norms, the adoption of body piercing and tattooing represents a significant shift. While conservative values remain prominent, increasing globalization, exposure to diverse cultures, and the widespread use of digital platforms have contributed to changing perceptions. This shift is especially evident among young adults, who are more likely to embrace body art as a form of personal expression. This trend holds particular significance in the Saudi context, where it represents a profound departure from traditional and religious norms that have historically discouraged such modifications. The convergence of rapid globalization, pervasive social media influence, and a regulatory vacuum governing the safety and hygiene of body art establishments creates a critical public health imperative (4–6).

However, this growing trend raises important questions regarding awareness of associated health risks, safety practices, and the sources of information that influence these decisions. Despite the growing body of international literature on body art practices, evidence from Saudi Arabia remains limited and fragmented, with existing studies often focusing on specific subgroups or isolated aspects such as knowledge or attitudes without integrating practices and reported complications (7). Furthermore, few nationwide studies have simultaneously examined awareness, behaviors, and health outcomes associated with both tattooing and body piercing within a single framework (8, 9). This gap underscores the need for comprehensive, population-based research that captures the multidimensional nature of body art practices in the Saudi context, which the present study aims to address.

Body piercing and tattoos involve invasive procedures that, if not performed under proper hygiene and safety standards, can result in significant health complications. In addition, improper aftercare can exacerbate these risks, leading to prolonged healing times and other adverse outcomes. With respect to tattoos, the incidence of dermatological complications ranges from 2% to 43% (10–12), surpassing that of systemic complications. A survey of 3,411 individuals indicated that 2,302 (67.5%) experienced cutaneous issues, compared with only 225 (6.6%) who reported extracutaneous complications (11). Tattooing can lead to significant dermatological issues, including hypersensitivity-induced rashes, allergic contact dermatitis, lichenoid eruptions, granulomatous skin diseases, and hypertrophic scars and keloids (13). Additionally, cutaneous infections can rapidly develop post-tattooing, encompassing viral infections such as verruca vulgaris, molluscum contagiosum, hepatitis B virus (HBV) and C virus (HCV), herpes simplex, or human immunodeficiency virus (HIV); bacterial infections including Streptococcus, Staphylococcus, Pseudomonas aeruginosa, cutaneous tuberculosis, syphilis, pyoderma, and dysbacteriosis; and fungal infections such as dermatophytosis and sporotrichosis (13, 14). Among these complications, allergic and photoallergic reactions, infections, and the Koebner phenomenon are the most frequently reported (15).

The occurrence of complications related to body piercings is notably high, with a prevalence of approximately 30% (16, 17). Among these complications, localized infections are the most frequent, accounting for 77% of cases (17, 18). It is estimated that localized diseases affect around 20% of body piercings (19). The most common pathogens responsible for these infections include Staphylococcus aureus and Group A Streptococcus in lobular ear piercings, and Pseudomonas aeruginosa in cartilage ear piercings (19, 20). Symptoms of ear-piercing infections include erythema, pain, and discharge of blood or pus. Despite the health implications, there is limited data on the level of knowledge young adults have about these risks, especially in the Saudi context.

This study examined awareness, attitudes, and practices related to body piercing and tattooing among individuals in Saudi Arabia, with a primary focus on younger populations. Tattooing and body piercing were considered within a unified framework as both represent prevalent forms of body art that share common social, cultural, and behavioral influences, particularly among youth (9). In addition, both involve invasive procedures associated with potential health risks, including infectious and non-infectious complications, and are typically performed in similar settings with comparable hygiene and regulatory considerations (21). Studying them together enabled a comprehensive evaluation of risk awareness and behaviors while allowing for distinction between practices where necessary (22).

The study aimed to assess participants' knowledge of health risks, motivations for engaging in body art, and preventive measures undertaken to avoid complications, while also examining the influence of social, cultural, and religious factors, as well as the role of education and public health initiatives in shaping these behaviors. This study provides original evidence from a large nationwide sample in Saudi Arabia by integrating behavioral practices, health-risk awareness, perceived complications, and demographic associations related to body art within a single analytical framework. The novelty of the study lies in its comprehensive evaluation of both infectious and non-infectious risks associated with body modification practices in a sociocultural context where such behaviors remain underexplored. The findings provide region-specific evidence that may inform future public health policies, educational interventions, and regulatory strategies regarding body art practices. To provide clarity and focus, these objectives were operationalized into the following research questions: (1) What is the level of awareness regarding health risks associated with tattoos and body piercings? (2) What attitudes and perceptions are held toward body art practices in the Saudi context? (3) What practices and behaviors are associated with body piercing and tattooing, including decision-making and aftercare? and (4) What types and frequencies of complications are reported, and how are these associated with demographic factors? These questions guided the study design, data collection, and analysis, and informed the development of targeted interventions to promote safer practices amid the increasing prevalence of body art in Saudi Arabia.

## 2 Materials and methods

### 2.1 Study design and ethics

This study employed a cross-sectional observational design with a descriptive, quantitative approach. This study was conducted in accordance with the Declaration of Helsinki and approved by the Research Ethics Committee of King Khalid University (Approval No. ECM#2023-3318, dated 19 December 2023).

### 2.2 Study area and setting

The survey was conducted nationwide across all regions of Saudi Arabia. Participants were recruited through an online questionnaire accessible via Google Forms. This platform choice ensured broad accessibility and convenience for participants. The survey targeted young adults who had undergone facial piercings and/or tattoos.

### 2.3 Data collection and source

Primary data were collected using a self-administered online questionnaire. Participants received a link to the questionnaire hosted on Google Forms. The accompanying cover letter provided an overview of the study's objectives, the principal investigator's contact information, and details on participant rights and confidentiality. The questionnaire was available in both Arabic and English. Measures were implemented to prevent duplicate submissions and ensure that no identifying information was collected, thus maintaining participant confidentiality. Participants were informed of their right to withdraw from the study at any time. For participants under the age of 18 years, additional ethical considerations were applied in accordance with institutional review board requirements. Participation was voluntary and anonymous, and the questionnaire was designed to pose minimal risk. In line with approved ethical procedures for online survey research involving minors, completion of the questionnaire was considered assent to participate, and the study introduction provided information to ensure that participants understood the purpose, confidentiality, and voluntary nature of the study.

### 2.4 Sample size calculation

The minimum required sample size was calculated using a prevalence estimate of 41.8% from a similar study (23), with *Z* = 1.96 at a 95% confidence level and a precision level of 5%, yielding a minimum sample of 373 participants. To improve representativeness and ensure adequate power for subgroup analyses across demographic categories, recruitment was continued beyond this minimum requirement through nationwide online distribution. The final analytic sample, therefore, comprised 1,472 complete responses. This number reflected the total number of eligible participants who completed the survey and was not the direct result of a 20% non-response adjustment to the minimum calculated sample size.

### 2.5 Sampling method and criteria

A purposive non-probability sampling approach was employed to recruit participants based on predefined inclusion and exclusion criteria. Data were collected באמצעות an online survey to enable efficient access to a geographically diverse population across all regions of Saudi Arabia, which was appropriate given the exploratory nature of the study and the absence of a centralized sampling frame for individuals engaged in body art. While this approach facilitated broad and rapid data collection, it may have introduced selection bias, as participation depended on internet access and willingness to respond, potentially leading to overrepresentation of more engaged or health-aware individuals and limiting generalizability, particularly to rural or less digitally connected populations.

The study population included individuals aged 12–50 years residing in Saudi Arabia, regardless of prior experience with body piercing or tattooing. Although the survey was intended to target individuals with body art, participation was not restricted in practice, and responses from both individuals with and without prior body modification were included. Participants without body art were retained to assess awareness, attitudes, and future intentions, whereas analyses of practices and complications were limited to those who reported prior piercing and/or tattooing.

The inclusion of a broad age range was intended to capture variability across adolescents, young adults, and older adults, enabling comparative analysis of age-related differences in awareness, attitudes, and behaviors. Individuals aged 12–17 years were included to reflect early exposure to body piercing practices. Minors participated in accordance with institutional ethical approval, with participation voluntary and anonymous. Although the term “young adults” is used throughout the manuscript to reflect the predominant demographic trend, it does not define strict eligibility criteria but rather the primary analytical focus. Individuals younger than 12 years and those unable to complete the survey due to critical illness were excluded to ensure data quality and relevance to the study objectives (24).

### 2.6 Research instrument and validation

The study employed a web-based survey comprising 20 questions to assess participants' perceptions and knowledge of the risks associated with facial piercings and tattoos. The validated questionnaire, adapted from “Perception and Knowledge of Oral and Facial Piercings among Dental Students: Web-based Survey” (23), was developed with the original authors' permission to align with the study's objectives and facilitate exploration of participants' understanding of these risks. It included four demographic questions, one perception question, and six knowledge questions. It was designed to be completed in 8–10 min and featured close-ended, yes/no, yes/no/do not know, and multiple-response formats. For females, the term “piercing” excluded earlobe piercings, while for males it included them. The survey covered general information on oral and facial piercings, reasons for these practices, potential complications, and awareness of health risks. In this manuscript, “body art” is used as an overarching term encompassing both tattooing and body piercing. These categories are presented separately where appropriate due to differences in procedure, risk profiles, and associated complications. Additionally, anatomical terms for piercing locations (e.g., earlobe, cartilage, nose) are used deliberately to reflect clinically relevant distinctions, particularly regarding complication rates and infection risks. This structured use of terminology is maintained throughout to ensure both clarity and scientific accuracy. Responses were de-identified, and only complete surveys were included in the analysis. The validated questionnaire was translated into Arabic to ensure accessibility for the target population. The translation process involved forward translation by two independent translators, reconciliation, and back translation to verify conceptual accuracy and cultural relevance. The Arabic version was then reviewed by a panel of dermatology and public health experts for content validity and pilot-tested with a sample of 30 individuals for clarity and reliability.

### 2.7 Statistical analysis

The statistical analysis of the data included descriptive statistics, where frequencies and percentages were calculated for each category of demographic variables, including age, level of education, gender, and region. The awareness of risks associated with body piercing and tattoos was examined, where frequencies and percentages were calculated for each question. The distribution of responses for each question was visualized using bar plots. Additionally, the frequency and percentage of participants who had piercings were calculated and presented in a bar plot, and the common locations of piercings were examined. Furthermore, the frequency and percentage of participants who would consider getting a piercing or tattoo in the future were calculated and presented in bar plots. Inferential statistics were also employed to examine the associations between the responses to the questions and the demographic variables. The Chi-square test was used to investigate the association between each question and age category, and the results were presented in a table. The Chi-square test was also used to examine the association between each question and gender, and the results were presented in a separate table. The *p-values* were calculated, and significant associations (*p* < 0.05) were reported. All statistical analyses were performed using Statistical Packages for Social Sciences (SPSS) version 28. All survey responses were screened for completeness before analysis, and questionnaires with substantial missing data were excluded from inferential testing. Categorical variables were summarized using frequencies and percentages, while subgroup comparisons were conducted using Pearson's chi-square test to evaluate associations between demographic characteristics and body art-related attitudes and practices. Statistical outputs were independently reviewed by the research team to ensure analytical consistency and accuracy.

## 3 Results

### 3.1 Demographic characteristics

The majority of participants (45.65%) were between 19 and 24 years old, followed by those over 35 (36.62%), 25–34 (12.77%), and under 35 (4.96%). In terms of education, most participants held a bachelor's degree (61.21%), while 23.78% had a high school education or lower, 13.72% had some college or an associate degree, and 1.29% had elementary education. The sample was predominantly female (77.11%), with 22.89% male. Regionally, the majority of participants were from the Southern region (43.55%), followed by Western (30.16%), Central (10.73%), Eastern (7.88%), and Northern (5.37%), with 2.31% not reporting their region (Table 1).

**Table 1:** Demographic data, including age category, level of education, gender, and region.

Variable	Category	*n* (%)
Age	19–24	672 (45.65)
	25–34	188 (12.77)
	< 35	73 (4.96)
	>35	539 (36.62)
Education	Bachelor degree	901 (61.21)
	Elementary	19 (1.29)
	High school or lower	350 (23.78)
	Some college or an associate degree	202 (13.72)
Gender	Female	1,136 (77.11)
	Male	336 (22.89)
Region	Central	158 (10.73)
	Eastern	116 (7.88)
	Northern	79 (5.37)
	Not reported	34 (2.31)
	Southern	641 (43.55)
	Western	444 (30.16)

### 3.2 Awareness of health risks

The risks associated with body piercing and tattooing are presented in Table 2. The majority of participants (77.31%) reported being aware of the health risks associated with body art practices, while 22.69% were not. Most participants (73.37%) believed there are risks associated with undergoing tattooing or piercing, with 22.49% unsure and 4.14% not believing there are risks. Regarding disease transmission, 67.87% of participants believed tattoos and piercings can transmit infectious diseases, and 37.37% believed they can transmit non-infectious diseases. However, 77.45% of participants did not think the places and instruments used for body art are always safe in terms of health and hygiene. Additionally, 30.84% of participants did not think it was possible to completely remove a tattoo, and 49.25% believed the effect of piercing is permanent. Overall, the results suggest that while most participants are aware of the risks associated with body art practices, there is still a need for education on the specific risks and consequences.

**Table 2:** Awareness of risks associated with body piercing and tattoos.

Question	Answer	*n* (%)
Are you aware of the health risks associated with body art practices such as tattooing and piercing?	No	334 (22.69)
	Yes	1,138 (77.31)
Do you believe there are risks associated with undergoing tattooing or piercing?	No	61 (4.14)
	Unsure	331 (22.49)
	Yes	1,080 (73.37)
Can tattoos and piercings transmit infectious diseases?	No	85 (5.77)
	Unsure	388 (26.36)
	Yes	999 (67.87)
Can tattoos and piercings transmit non-infectious diseases?	No	303 (20.58)
	Unsure	618 (42.05)
	Yes	551 (37.37)
Do you think the places and instruments used for body art are always safe for health and hygiene?	No	1,141 (77.45)
	Unsure	235 (15.96)
	Yes	96 (6.59)
Is it possible to completely remove a tattoo?	No	454 (30.84)
	Unsure	679 (46.13)
	Yes	339 (23.03)
Is the effect of piercing permanent?	No	123 (8.36)
	Unsure	624 (42.39)
	Yes	725 (49.25)

### 3.3 Prevalence and types of body art

The locations of piercings are presented in Table 3. The majority of participants (83.69%) reported having an ear piercing, followed by the nose (8.77%), navel (3.54%), and lips (2.15%). Other locations, including the cheek, eyebrow, tongue, and uvula, were less common, each accounting for 0.46% of total piercings. These results suggest that ear piercings are by far the most popular type of piercing among the participants, with other locations being relatively less common.

**Table 3:** Frequency and distribution of piercing locations among participants.

Location	*n* (%)
Cheek	3 (0.46)
Ear	544 (83.69)
Eyebrow	3 (0.46)
Lips	14 (2.15)
Navel	23 (3.54)
Nose	57 (8.77)
Tongue	3 (0.46)
Uvula	3 (0.46)

### 3.4 Attitudes and practices

**Figure 1:**
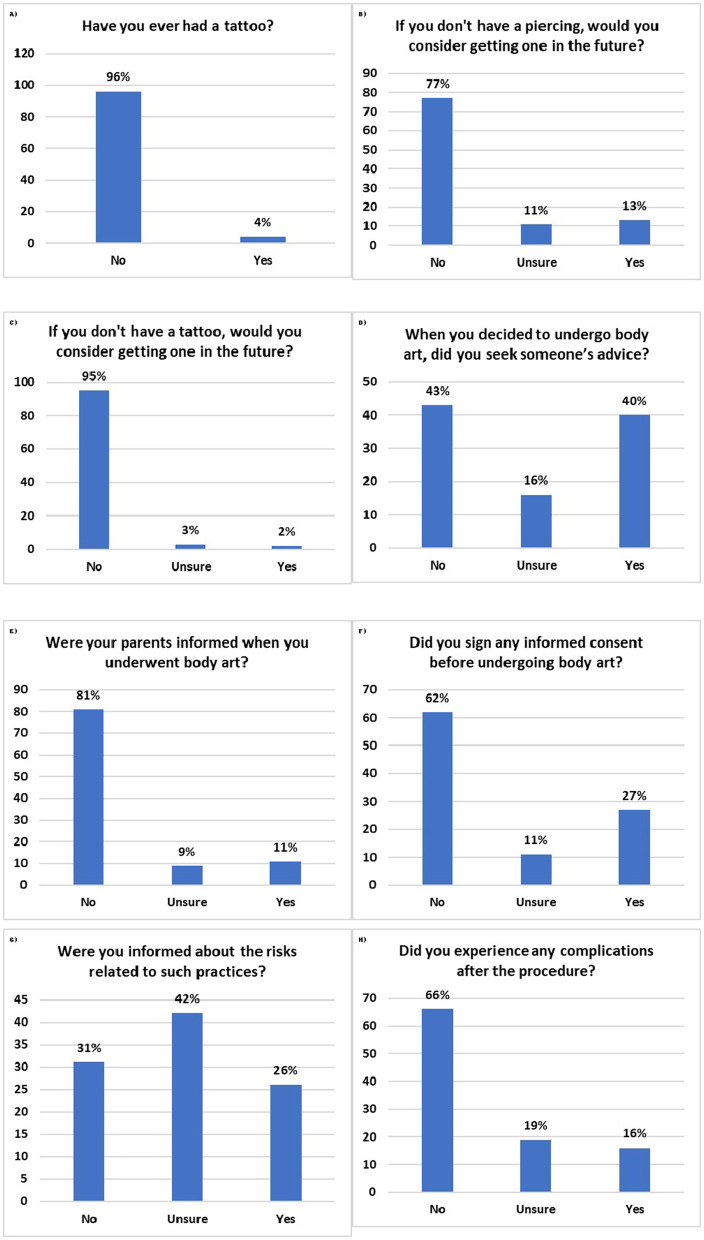
Participants' attitudes and practices regarding body art, including tattoo ownership **(A)**, willingness to get a piercing in the future **(B)**, willingness to get a tattoo in the future **(C)**, tendency to seek advice before undergoing body art **(D)**, parental involvement in body art decisions **(E)**, signing of informed consent prior to the procedure **(F)**, awareness of associated risks **(G)**, and experience of post-procedure complications **(H)**.

The survey results revealed participants' varied attitudes and experiences with body art (Figure 1). A significant majority expressed no interest in acquiring body art: 96% reported no tattoos, and 95% of those without tattoos said they would not consider getting one in the future. Similarly, 77% of participants without piercings were not interested in obtaining one. When it came to seeking advice before undergoing body art, participants were nearly evenly split: 43% did not seek advice, 40% did, and 16% were unsure. Additionally, 81% did not inform their parents before undergoing body art, indicating low parental involvement in these decisions. Regarding procedural protocols, only 27% signed informed consent, and 31% were aware of the risks associated with body art, highlighting a gap in informed practice. Finally, complications were relatively uncommon, with only 16% of participants reporting any issues post-procedure.

### 3.5 Associations with demographics

Associations between questions and age groups were examined using the chi-square test (Table 4). Results showed significant associations between age groups and responses to questions about the acceptability of mouth and facial piercings in society (*p* < 0.001), the risks or complications associated with oral and facial piercings (*p* < 0.001), and the necessity of parental consent for oral and facial piercings (*p* < 0.001). Additionally, a significant association was found between age groups and the reasons for tattooing or piercing (*p* < 0.001). Specifically, younger age groups (19–24 and 25–34) were more likely to consider mouth and facial piercings acceptable in society. In contrast, older age groups (>35) were more likely to consider them insufficient. Similarly, younger age groups were more likely to be unsure about the risks or complications associated with oral and facial piercings, whereas older age groups were more likely to believe there are risks. Furthermore, younger age groups were less likely to consider parental consent necessary for oral and facial piercings, whereas older age groups were more likely to consider it necessary. Finally, the reasons for tattooing or piercing varied across age groups, with younger age groups more likely to cite cosmetics or no reason, and older age groups more likely to cite the pursuit of fashion.

**Table 4:** The association between questions and age groups using chi-square test.

Questions	Answer	Age					*Chi-square p-value*
		19–24	25–34	<35	>35	Total	
		*n* (%)	*n* (%)	*n* (%)	*n* (%)	*n* (%)	
Do you think that mouth and facial piercings are acceptable in society?	No	311 (66.31)	110 (75.34)	31 (58.49)	377 (88.50)	829 (75.78)^*^	**< 0.001**
	Unsure	115 (24.52)	23 (15.75)	15 (28.30)	38 (8.92)	191 (17.46)	
	Yes	43 (9.17)	13 (8.90%)	7 (13.21)	11 (2.58)	74 (6.76)	
Are there risks or complications associated with oral and facial piercings?	No	311 (66.31)	110 (75.34)	31 (58.49)	377 (88.50)	829 (75.78)	**< 0.001**
	Unsure	115 (24.52)	23 (15.75)	15 (28.30)	38 (8.92%)	191 (17.46)	
	Yes	43 (9.17)	13 (8.90)	7 (13.21)	11 (2.58)	74 (6.76)	
Do you think parental consent for oral and facial piercings is necessary?	No	141 (29.81)	30 (20.98)	12 (23.08)	36 (8.35%)	219 (19.93)	**< 0.001**
	Yes	332 (70.19)	113 (79.02)	40 (76.92)	395 (91.65)	880 (80.07)	
What is the reason for your tattooing or piercing?	Cosmetics	168 (45.41)	34 (31.48)	15 (36.59)	92 (32.06)	309 (38.34)	**< 0.001**
	No reason	167 (45.14)	51 (47.22)	20 (48.78)	84 (29.27)	322 (39.95)	
	Pursuit of fashion	35 (9.46)	23 (21.30)	6 (14.63)	111 (38.68)	175 (21.71)	

^*^Percentages are based on respondents who answered all questions in the analysis. Sample sizes vary due to item non-response. Bold values indicate statistically significant associations (*p* < 0.05) based on the Chi-square test.

**Figure 2:**
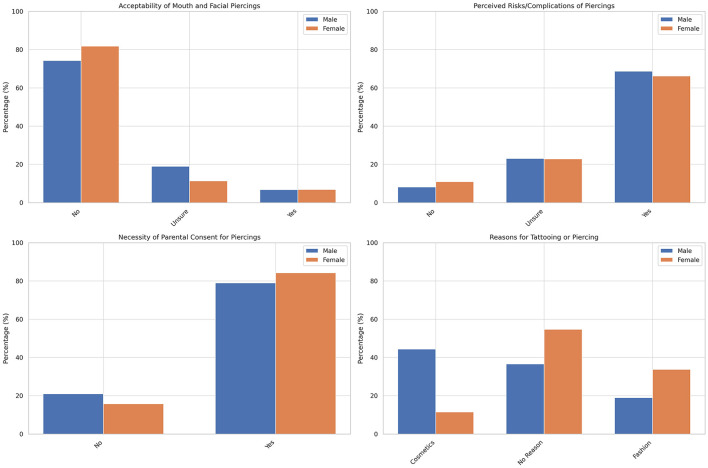
Gender-based associations with attitudes and practices toward body art: chi-square analysis.

Associations between questions and gender were examined using the Chi-square test (Figure 2). The results showed a significant association between gender and responses to the question about the acceptability of mouth and facial piercings in society (*p* = 0.027), with a higher proportion of female participants (81.82%) reporting that they do not think mouth and facial piercings are acceptable compared with male participants (74.26%). A significant association was also found between gender and the reasons for tattooing or piercing (*p* < 0.001), with a higher proportion of female participants (54.73%) reporting no reason for their tattoo or piercing than male participants (36.63%). Additionally, a higher proportion of male participants (68.74%) reported risks or complications associated with oral and facial piercings than female participants (66.21%), but this difference was not statistically significant (*p* = 0.427). No significant associations were found between gender and responses to questions about parental consent for oral and facial piercings (*p* = 0.082). These results suggest that gender is associated with the acceptability of mouth and facial piercings in society and with the reasons for tattooing or piercing, but not with perceptions of risks or complications associated with oral and facial piercings or with the necessity of parental consent.

### 3.6 Reported complications

**Figure 3:**
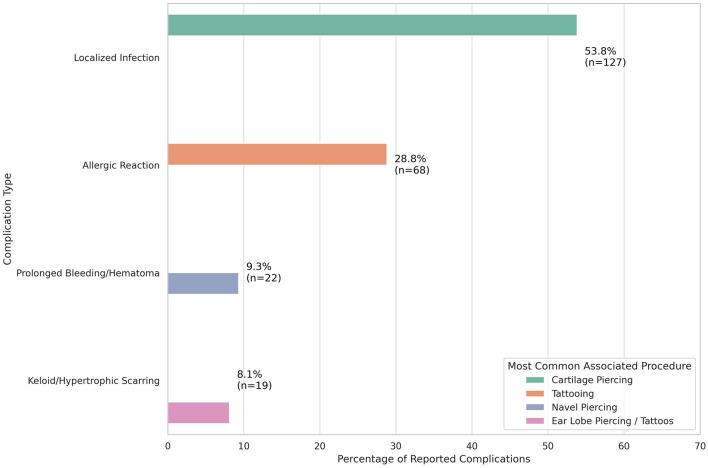
Distribution of reported complications from body art procedures by type and associated procedure.

Among the total sample of 1,472 participants who had undergone body modification procedures, a notable proportion (16%, *n* = 236) reported at least one complication. The nature and frequency of these complications varied significantly by the type and location of the body art. The distribution of complications was strongly associated with the location of the body art. Piercing-related complications were most frequently reported for cartilage piercings (e.g., upper ear, nose) (68% of piercing complications), primarily infections characterized by erythema, pain, and purulent discharge. In contrast, standard earlobe piercings, while the most common procedure (83.69%), had a comparatively low complication rate (12% of piercing complications), mostly minor infections or keloid formation in predisposed individuals (Table 5 and Figure 3). Among participants with tattoos (4%, *n* = 59), 31% (*n* = 18) reported complications. The most frequent issue was an allergic reaction (66.7% of tattoo complications), often characterized by persistent itching and raised skin, particularly in areas with red or green pigment. A smaller subset reported infections (22.2%) and issues with tattoo fading or blurring (11.1%). A Chi-square test of independence revealed a significant association between the procedure location and the likelihood of experiencing a complication (χ^2^ = 38.4, *p* < 0.001). Cartilage piercings and tattoos were 3.2 times [95% CI (2.1, 4.8)] more likely to be associated with a reported complication than earlobe piercings. Furthermore, among those who experienced complications, only 35% sought professional medical care, while the majority (65%) resorted to self-treatment with over-the-counter antiseptics or home remedies.

**Table 5:** Type and frequency of reported complications (*n* = 236).

Complication type	*n*	% of complications	Most common associated procedure
Localized infection (pain, redness, pus)	127	53.8%	Cartilage piercing (ear, nose)
Allergic reaction (itching, rash)	68	28.8%	Tattooing (various colors)
Prolonged bleeding/Hematoma	22	9.3%	Navel piercing
Keloid or hypertrophic scarring	19	8.1%	Ear lobe piercing, tattoos
Total reported complications	236		

Participants could report more than one complication.

## 4 Discussion

The findings of this study shed light on the growing trend of body art, including piercings, tattoos, and micro-blading, among young Saudi adults. This study addresses a notable knowledge gap regarding awareness of the associated risks and complications, particularly in a region where cultural, regulatory, and public health considerations are evolving. The absence of official records on complications from tattoos and piercings in Saudi Arabia complicates efforts to assess and address public health risks.

### 4.1 Awareness of risks

The majority of participants in this study (77.31%) reported awareness of the health risks associated with body art practices, such as tattoos and piercings. This level of awareness is encouraging; however, a significant gap remains, as only 37.37% acknowledged the risk of non-infectious complications. This finding aligns with previous studies, including those by Quaranta et al. (8) and Gallè et al. (28), which also reported significant knowledge gaps regarding the health risks of body art among young adults. Consistent with our findings, these studies found that although participants recognized the potential risks, their understanding of specific complications remained limited. This pattern may be attributed to the nature of information sources accessible to young individuals: general awareness of risks is often disseminated through social media and peer networks, whereas detailed medical knowledge of specific complications is less frequently communicated (8). Additionally, cultural sensitivities surrounding body art in Saudi Arabia may limit open discussion and formal education on the topic, thereby contributing to superficial awareness without in-depth understanding. These findings have important public health implications, as partial knowledge may lead individuals to underestimate certain risks or fail to adopt appropriate preventive measures (8). Addressing this gap requires targeted educational strategies that move beyond general awareness and emphasize specific complications, safe practices, and the importance of professional standards in body art procedures.

The study by Kinkar et al. (6) also supports these observations, noting that only 39.6% of participants in Saudi Arabia had good knowledge of the hazards associated with tattoos and piercings. The relatively low awareness of non-infectious risks underscores the need for improved public education on the diverse complications that can arise from body art practices. The limited awareness of non-infectious complications, coupled with the low proportion of participants who report having provided informed consent prior to procedures, raises important concerns about the quality of decision-making and risk communication in body art practices (25). These findings suggest that individuals may undergo procedures without a comprehensive understanding of potential long-term consequences, including allergic reactions, scarring, and other non-infectious outcomes (25). The absence of adequate informed consent further indicates potential gaps in professional standards and regulatory oversight within body art settings (26). Collectively, these issues may contribute to preventable adverse outcomes and highlight the need for stricter enforcement of consent protocols and standardized risk disclosure practices (26). Strengthening these aspects is essential to ensure that individuals can make fully informed decisions and to reduce the burden of avoidable complications associated with body art (27).

### 4.2 Safety concerns and regulatory gaps

A significant portion of our participants (77.45%) expressed concerns about the safety of the places and instruments used for body art. This concern is consistent with findings from Gallè et al. (28), who highlighted regional discrepancies in risk awareness and hygiene standards. In Saudi Arabia, the absence of formal regulations and health permits for body art practitioners likely contributes to the increased risk of complications. The lack of oversight raises concerns about hygiene standards and the adequacy of infection control measures, as also noted by Tamene et al. (29), who emphasized the importance of experience and proper training in ensuring safe body modification practices. Binkhamis et al. (4) further underscore the need for better regulation, noting that cartilage piercings are particularly susceptible to infection and have a higher complication rate than earlobe piercings. Their study highlighted that the use of piercing guns, which unlicensed practitioners often employ, contributes to higher infection rates, reinforcing the necessity for stricter regulation and professional standards.

### 4.3 Perceptions and practices

This study found that ear piercings were the most common form of body art, with 83.69% of participants having them, reflecting global trends where ear piercings are widely accepted and practiced. However, cultural factors also influence preferences for other types of piercings, such as those on the nose or navel, which are less prevalent.

Interestingly, the study revealed a low interest in future tattoos or piercings among participants without prior experience. Specifically, 95% of participants without tattoos and 77% without piercings indicated they would not consider getting either in the future. This suggests that body art is often a trend among those who already engage in it, rather than a widespread desire among the general population. Similar to our findings, Blázquez Abellán et al. (30) noted that social drivers such as aesthetics, self-expression, and peer influence often outweigh health considerations, especially among women and younger individuals.

### 4.4 Tattoo regret and complications

Tattoo regret and associated complications are critical issues in body art practices. Mitwalli et al. (5) reported a high level of tattoo regret (58%) among individuals in Saudi Arabia, with pruritus being the most common complication. This finding emphasizes the importance of careful consideration and informed consent before undergoing tattoo procedures, as dissatisfaction and health risks can have lasting consequences.

### 4.5 Educational needs and public health implications

Based on the findings, several recommendations can be made to improve awareness and safety regarding body art practices. First, there is a clear need for enhanced educational initiatives targeting young adults, emphasizing not only the general risks but also specific complications related to body piercing and tattoos. Public health campaigns should be tailored to address knowledge gaps, particularly regarding non-infectious complications and the safety of body art procedures. Health professionals and regulatory bodies should collaborate to develop and enforce standards for hygiene and safety in body art practices, ensuring that practitioners follow best practices to minimize risks. Additionally, educational programs should include guidance on the importance of informed consent and the potential long-term effects of body art. Engaging with local communities and cultural leaders may also help bridge cultural gaps and support the acceptance of safe body art practices. Future research should explore the effectiveness of these educational interventions and investigate other factors influencing body art practices, such as cultural beliefs and socioeconomic status.

### 4.6 Strengths and limitations

This study offers valuable insights into awareness and practices related to body art among young adults in Saudi Arabia. A significant strength of the research is its comprehensive approach, which includes a large, diverse sample across various regions of Saudi Arabia, thereby enhancing the generalizability of the findings. The use of an online questionnaire facilitated data collection from a broad audience, ensuring inclusivity and ease of participation. Additionally, the study's focus on both body piercing and tattoos, along with its detailed analysis of associated risks and complications, provides a nuanced understanding of the topic. The inclusion of demographic variables, such as age and gender, enables a thorough exploration of how these factors shape perceptions and practices regarding body art. However, the predominance of female participants in the sample may have influenced the overall findings, particularly regarding attitudes, awareness, and practices associated with body art. Given that previous research suggests gender differences in motivations, risk perception, and engagement with body art, the overrepresentation of females may limit the generalizability of the results to the broader population. This imbalance should be considered when interpreting the findings, and future studies should aim for a more balanced gender distribution to enable more robust comparative analyses. The statistical methods employed, including the Chi-square test for associations, strengthen the validity of the results and provide a foundation for future research. This study has several limitations. Chief among them is the use of a purposive sampling strategy, which inherently introduces selection bias. Although this method was effective in recruiting a sufficient number of participants with body art, it limits the generalizability of the findings to all young adults in Saudi Arabia. The sample was self-selected, likely over-representing individuals more engaged with or positive toward body art, and those with internet access, potentially under-representing more conservative viewpoints or those from rural areas. The reliance on self-reported data could also introduce bias, as participants might underreport or overreport their practices and experiences. Additionally, the cross-sectional nature of the study limits the ability to draw causal conclusions about the relationships between awareness, practices, and demographic variables.

## 5 Conclusions

This study identified important gaps in knowledge and practices related to body art among participants in Saudi Arabia. Although a majority of participants demonstrated general awareness of health risks (77.31%), detailed understanding remained limited, particularly regarding non-infectious complications (37.37%). In addition, procedural practices were suboptimal, as evidenced by a low rate of informed consent (27%) and limited engagement with professional guidance prior to body art. The high prevalence of ear piercings (83.69%), contrasted with higher complication rates associated with cartilage piercings and tattoos, further highlights the need for site-specific risk awareness.

These findings underscore the need for targeted and structured public health interventions that emphasize comprehensive education on both infectious and non-infectious risks, as well as the importance of safe practices and aftercare. Regulatory efforts should focus on establishing and enforcing standardized guidelines for hygiene, practitioner training, and mandatory informed consent procedures in body art settings. Public health campaigns should also leverage commonly used information channels, such as social media, to disseminate accurate and accessible information. Future research should adopt longitudinal designs to better assess causal relationships between awareness, behaviors, and health outcomes. Additionally, qualitative studies are needed to explore the social and cultural factors influencing body art decisions. Ensuring more demographically balanced and representative samples will further enhance the generalizability of findings and support the development of more effective, evidence-based interventions.

## Data Availability

The original contributions presented in the study are included in the article/Supplementary material, further inquiries can be directed to the corresponding author.
